# Dr. L. M. Guilding on the Effect of the Poor-Law Orders

**Published:** 1914-03-14

**Authors:** 


### NON-SEPARATED INFIRMARIES.

### Dr. L. M. Guilding on the Effect of the Poor-Law Orders.

The workhouse sick wards of the parish of Reading form one of the largest non-separated infirmaries in the country, and this fact provides a special reason why the new Poor-Law Orders, which come into force on March 31, can be illustrated to advantage in the light of its experience and work. With this end in view our Commissioner visited Reading and, through the courtesy of Dr. L. M. Guilding, learnt from him how the new Orders are likely to make for efficiency, and indirectly what is likely to be the future of the smaller rural workhouses in so far as they deal with the treatment of the sick.

### Federated Poor-Law Hospitals.

"I have been here for twenty years," Dr. Guilding began, "and have seen great changes; for instance, though the number of beds has not actually increased, there are now nineteen nurses where there were formerly three, and an infirm block which has been built of late years has helped in the classification of the sick and made us, with our 180 beds, including those for mental cases, one of the largest non-separated institutions in the country. It is hardly too much to say that up to ten years ago our buildings were a model to Poor-Law infirmaries in the country; and though for the last few years in certain respects newer institutions may be leaving us a little behind, our administrative system is in the main identical with that laid down by the new Poor-Law Orders."

"A House Committee is one of the new departures?"

"We have had one here for some years, and I think from experience that it makes for efficiency. A committee is always better than a board, because a board is too cumbersome, and in my opinion the ideal committee is a committee of one, which is, of course, what a medical superintendent of a separated infirmary amounts to. Of the effect of this House Committee on the small rural workhouses I am unable to speak, but I know enough of their condition to feel sure that the only possible way of dealing with them is by centralisation. They should be federated into Poor-Law hospitals, and serve recognised areas. This change will have to come one day, but it will not be in my time. There is no other way of making the standard of the infirmaries uniform throughout the country."

### The Effect of the Record Papers.

"What do you think of the record-papers on which the new Order insists?"

"Men who have done nothing hitherto will not like it, but the change is, of course, on the right lines. It will add to the work of those who have only accepted workhouse appointments to keep the field free for themselves, and have cared nothing much for the work for which, as the Royal Commission stated, such miserably inadequate pay is offered. But there is always this to be remembered: non-resident doctors cannot be made, nor should they be expected to keep elaborate notes. The keen and sensible among them invent for themselves a series of terms, and this series becomes sufficiently elastic to cover all ordinary contingencies. It all depends upon the individual. Some men have no faculty for compression, but it is inevitable that in contract work, and especially in badly-paid contract work, no amount of regulations can make a man spend more time on his work than he can help. The keen man gets over this difficulty by a system of hieroglyphics which form a record sufficiently intelligible to him. The man who only takes the work to keep away competitors, unless his salary is raised to make the extra clerical work worth while, or unless clerical assistance be provided, will not write much more than he has done hitherto. Doctors are proverbially averse to bookkeeping. As a matter of fact, a very great deal depends upon the superintendent nurse. Where, as here, for instance, she is an exceedingly capable woman, efficiency and care within the institution will be the result. The record-papers are on the right lines, but in a large institution like this they have already been largely adopted."

### A Grave Blemish and a Disgraceful System.

"Nothing is said in the Order against the Guardians contracting with the medical officer for the supply of medicines?"

"That is certainly a grave blemish. The present is a disgraceful system. A medical officer's salary ought to be absolutely irrespective of the supply of drugs. Supposing a case needs an expensive drug like '606,' for instance, a medical officer, in the present state of salaries, simply cannot afford to prescribe it. To expect him to do so is a form of sweating. Here, of course, that system has never obtained. My own salary has nothing to do with drugs or medical appliances. I order what I deem necessary, and the Guardians here have never made any difficulty."

"The salaries are not raised on account of the increased work?"

"The salaries question is a large one. The radical defect of the present system is that the Local Government Board prescribes and arranges the work, but merely sanctions and does not lay down the salaries. My own view is that the methods of the Prison Service should be followed. Have a Central Poor-Law Service on these lines, and there could not be the present anomaly of one authority laying down our duties and another arranging our pay. In Reading we have a very enlightened Board, but they are drawn from a large and prosperous commercial centre, while Boards in rural districts are largely drawn from farmers, who have the reputation of being a most parsimonious class."

### Medical Officer and Workhouse Master.

"The new Order gives precedence to the duties of the master over those of the medical officer. What are your views on this?"

"The master must have precedence. He is the only official with the power to punish, and the disciplinarian of an institution must, *ipso facto*, be its administrative head. You cannot get away from the fact that where one official, like the medical officer, is non-resident, and the other, like the master, resident, everything finally depends upon the resident man. If each has the interests of the institution at heart, and both work for the good of that—not for their own good, mind—everything will go smoothly. If not they can easily make the place a little hell for everybody. The stock ways of doing this are for the master to send for the medical officer unnecessarily, especially at night, and for the medical officer to upset the staff of the institution by ordering special diet, which will cause extra trouble in the kitchen, and the like.

Exactly the same situation may arise in the Prison Service between the governor and the prison doctor. You see an infirmary differs from a voluntary hospital in being unable to dismiss patients should they prove unamenable to discipline. You have to fall back on punishments and on moral control—a force as real as it is hard to define."

An Experience of Workhouse Masters.

"The new Order was criticised for giving power to the master to send an inmate, who arrives in the absence of the medical officer, to an appropriate ward, instead of keeping him in a receiving ward until the medical officer's arrival."

"A medical officer's views on such a criticism will depend entirely on what his experience of workhouse masters has been. I have found them as a class sensible men, and picked men, and those with whom I have hitherto had dealings, with one exception, have all had this character. Of course, the best men will not be found in the smaller rural workhouses—to raise the level of which, no doubt, the new Orders were devised—and of these I have had no experience. Our institution is a sort of half-way house between a non-separated and a separated infirmary, and certainly represents the very best of the former class. But this I will say. No doctor wishes to make unnecessary visits, and where the two men work together the power of the master to place new arrivals in what he believes to be a suitable ward should cause no real difficulty. The telephone provides the solution. Take my own case. One night a patient arrives here. The master telephones to me. I then ask him two questions. Has he any doubt as to his condition? Has any other doctor seen the case the same day? The master answers these questions in the light of his experience and common sense. Where the two officials work together the master's common sense provides sufficient practical guarantee that no grave mistake will be made. The master and I have always backed each other up in recommending improvements for the good of the institution."

The Value of the Lay Inspector.

"Are you one of those who regret the paucity of Local Government Board medical inspectors?"

"I can hardly say that personally I have had cause to do so. I have not had a recalcitrant Board of Guardians to deal with; and, again, where medical officers wish some reform to be backed up, much can be accomplished through the assistance of the lay inspector of the district. A medical officer who establishes personal relations with his local inspector can always rely upon a fair and unbiassed hearing, and frequently the inspector is gravely concerned at the condition of affairs which the medical officer desires to remedy. Here, again, the officials must work together. It is simply a method of going the right way to work."

The A B C of Administration.

"Was there much system when you first came here?"

"There were no books and no bed-cards. To-day when I call at the institution a row of report books are in front of me, each of which contains whatever of importance has happened in the institution during the past twenty-four hours. The night nurse of each block, for instance, writes a report of everything of importance that has happened in her wards during her period of duty. This is initialled *by the day nurse* as soon as she takes her place, so that no excuses can be made that she 'did not see' or 'did not know' anything specially requiring her attention. Anything of note is ticked by the superintendent nurse, and when the books come before me I in turn place a mark against the important points to show that I too have noticed them. In this way everyone is made responsible, and I have all the facts at my fingers' ends in about five minutes after my arrival.

A Short Way of Prescribing.

"Another example of method is the way in which my prescriptions for all these patients are carried out. I have a list of prescriptions, each of which is represented by a different letter of the alphabet, while certain modifications are connoted by a numeral following the letter. Prescription writing is, therefore, a very rapid process. The dispenser has, of course, a duplicate of my abbreviations, which serve for all the ordinary cases which my experience has led me to expect.

"As regards the patients, I see all new arrivals and urgent cases during my daily visit, and all others as a matter of routine. The new Orders, therefore, are not likely greatly to add to my purely medical work, though I shall probably have to secure further clerical assistance. On the whole, then, the new Orders will certainly make for efficiency, but as the system at Reading might well have served as a model on which they were based we shall not be so much affected by them as those institutions whose present condition they were specially designed to remedy."

